# The Mechanical Behavior of a Screwless Morse Taper Implant–Abutment Connection: An In Vitro Study

**DOI:** 10.3390/ma15093381

**Published:** 2022-05-08

**Authors:** Aimen Bagegni, Vincent Weihrauch, Kirstin Vach, Ralf Kohal

**Affiliations:** 1 Medical Center–University of Freiburg, Center for Dental Medicine, Department of Prosthetic Dentistry, Faculty of Medicine, University of Freiburg, Hugstetter Straße 55, 79106 Freiburg, Germany or vincentweihrauch@googlemail.com (V.W.); ralf.kohal@uniklinik-freiburg.de (R.K.); 2 Medical Center–University of Freiburg, Institute of Medical Biometry and Statistics, Faculty of Medicine, University of Freiburg, 79106 Freiburg, Germany; kv@imbi.uni-freiburg.de

**Keywords:** screwless, implant–abutment connection, conical, Morse taper, pull-off force

## Abstract

The use of screwless Morse taper implant–abutment connections (IAC) might facilitate the clinician’s work by eliminating the mechanical complications associated with the retention screw. The aim of this study is to evaluate the effect of artificial chewing on the long-term stability of screwless Morse taper IACs. Thirty-two implant abutments restored with an upper central incisor zirconia crown were used and divided into four groups according to the implant–abutment assembling manner (C1,H: screw retained (20 Ncm); C2: tapped; or C3: torqued (20 Ncm; the screws were removed before the dynamic loading)). All specimens were subjected to a cyclic loading (98 N) for 10 million chewing cycles. The survived samples were exposed to a pull-off force until failure/disassembling of the connection. All the samples revealed a 100% survival. Regarding the pull-off test, the screw-retained internal hexagonal IAC revealed significantly higher resistance to failure/disassembling (769.6 N) than screwless conical IACs (171.6 N–246 N) (*p* < 0.0001). The retention forces in the Morse taper groups were not significantly different (*p* > 0.05). The screw-retained hexagonal IAC showed the highest retention stability. The screw preload/retention in the conical IAC was lost over time in the group where the screws were kept in place during loading. Nevertheless, the screwless Morse taper IACs were stable for an extended service time and might represent a valid form of treatment for single-tooth replacement.

## 1. Introduction

The success of an implant treatment depends on the successful osseo- and soft tissue integration of the implant and also on the stability of the restoration (i.e., the implant–abutment–crown complex) [1,2,3,4,5,6]. Moreover, implant–abutment connections are considered a key factor of a successful implant therapy since they directly influence the long-term stability of the systems [7]. They represent the central stress resistance points of oral implants, as they must counteract the maximal and permanent masticatory forces. There are data that suggest that external implant–abutment connections are combined with higher rates of technical complications compared with internal connections [8,9,10]. However, a recent systematic review [8] reported a technical complication rate of 10.1% for internal implant–abutment connections and 12.4% for external implant–abutment connections. These differences between internal and external connections did not reach statistical significance (*p* = 0.431) [8].

Abutments serve as a link, connecting the oral implant and the prosthetic superstructure, and they are usually screw-retained to the implant. They can be customized or prefabricated by the manufacturer and made of various materials, such as titanium, gold, aluminum oxide, or zirconium oxide [9,11,12,13,14].

In general, there are two types of implant–abutment connections with two-piece implant systems (i.e., external and internal connections and the latter being divided into conical and butt–joint (internal hexagonal) connections). Internal conical or internal butt–joint connections show significantly less deformation and more stability compared with external connections [15]. Internal conical connections have been reported to have a better mechanical stability, namely, increased fatigue and fracture resistance compared with internal butt–joint connections [16,17,18].

Implant-supported superstructures may be either cement retained or screw retained. Screw-retained superstructures have the advantage that they can be easily removed upon demands, so technical and the biological complications can be managed more easily. Nevertheless, correct sagittal and transverse implant positioning in the hosting bone are of importance for this type of connections. Only a proper implant position and angulation enable the screw access opening in the anterior zone to be positioned in the invisible oral area [19]. On the other hand, as compared with screw-retained superstructures, the cement-retained restorations are considered to be easier to fabricate and less expensive and may be preferred to compensate for improperly inclined implants [20]. However, it is assumed that excess cement around dental implants is a potential risk factor/indicator for biological complications [20]. Nevertheless, in [21], the proportions of diseased implant sites showing excess cement varied considerably among studies, and thus, linking peri-implantitis to the postrestorative presence of submucosal cement is still inadequate.

However, the most common reported technical complications of implant-supported prosthetic constructions are screw loosening or screw fractures [8,22,23]. Pjetursson and his group (2018) reported a prevalence of screw loosening in external connections for implant-supported single crowns at 4.8% and that with internal connections at 1.2% over an observation period of 5 years [8]. The loss of screw torque might be caused by the axial and nonaxial forces subjected to the implant superstructure. The nonaxial forces might be provoked by the implant prosthetic part according to the lever principles. Nissan et al. (2011) reported that any forces applied on the implant superstructure are followed with tensile and compressive stresses at the position of the implant–abutment connections, resulting in micromovements between the different components (the implant and the abutment), which might be terminated with screw torque loss complications [23,24].

In an attempt to limit the fixation screw complications, an alternative to screw-retained implant systems (i.e., screwless Morse taper implant–abutment connections) has been manufactured [25,26]. This type of connections provides the required retention through the “cold welding” principle. The elastic deformation of both, the implant and the abutment, creates a high-tension force between them. The use of a screwless Morse taper implant–abutment connection might be useful to clinicians by eliminating the mechanical complications associated with the retention screw and giving an advantage in regard to the rehabilitation of the misaligned implants by avoiding a screw access canal (e.g., in the anterior region). It is thus useful to evaluate the fatigue behavior and the retention stability of screwless Morse taper implant–abutment connection designs using different insertion and retention methods in an in vitro environment. Therefore, the objective of this in vitro study was to evaluate the long-term fatigue and the retention stability of a Morse taper implant–abutment connection by applying dynamic chewing simulation. The null hypothesis is that both screw-retained and screwless implant–abutment connections show the same mechanical and retention stability.

## 2. Materials and Methods

### 2.1. Outline of the Study

**Figure 1:**
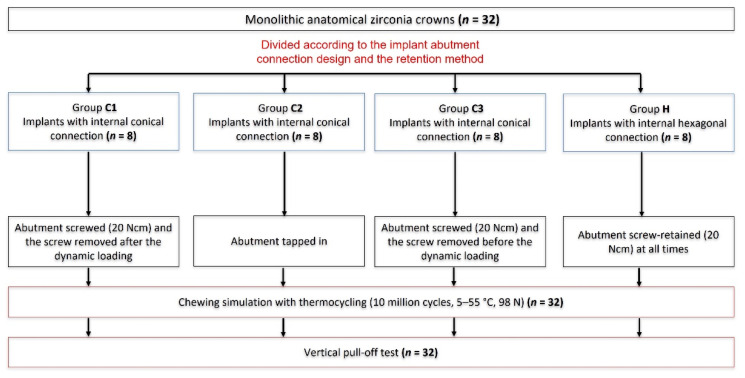
Setup of the study (H: internal hexagonal connection; C: internal conical connection).

For this in vitro study, a total of 32 samples consisting of 32 crowns made of zirconia (Dental Direkt GmbH, Spenge, Germany) mounted on two different types implant–abutment connections were evaluated. According to the implant–abutment assembly method, the samples were then categorized into four main groups. The first group of samples was screw-retained on titanium implants with an internal conical (C1) connection, and the screws were kept throughout the dynamic loading and were removed after dynamic loading before the pull-off test. The second group was a tapped-/knocked-in screwless internal conical (C2) connection (no screws were used for this group). The third group was screwless internal conical (C3) connection. The screws were used only to seat the abutments into the implants and removed before the dynamic loading. The fourth group (control group) was a screw-retained internal hexagonal (H) connection. Dynamic loading test in an artificial chewing simulator was applied to all samples. After chewing simulation, the specimens were investigated using a light microscope to observe any defects. The survived samples were subjected to pull-off test until the crowns or abutments were detached from the implants in a universal testing machine (Figure 1).

**Figure 2:**
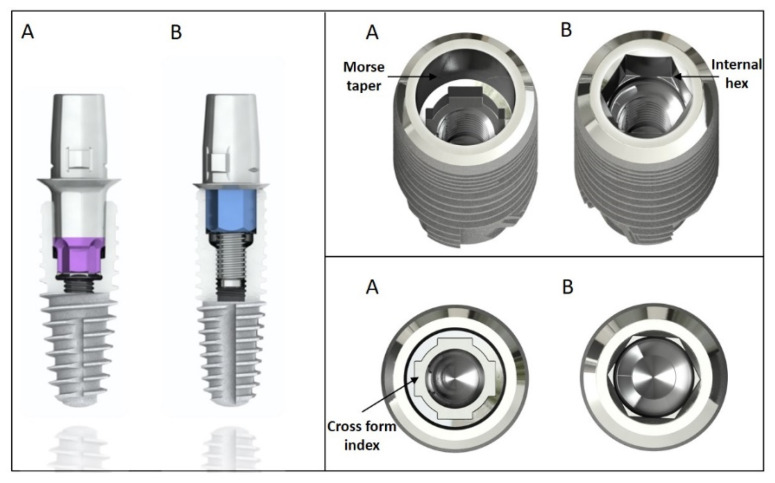
(**A**) Implant with an internal conical connection; (**B**) implant with an internal hexagonal connection.

The implants utilized in this in vitro study were made of titanium (Grade 4) and were 14.5 mm long and had a diameter of 4.2 mm. The implants had one of the two following internal connection characters:

- Groups C1–C3: Morse taper internal conical connection (SICvantage tapered, SIC Invent AG, Basel, Switzerland). A cone angle of 2.8° and a cone length of 3 mm; indexed with four grooves in a cross form (Figure 2A).
- Group H: Internal hexagonal connection (SIC tapered, SIC Invent AG) characterized with parallel-walled orientation surfaces (Figure 2B).

Prefabricated abutments were used as crown substructures. The following prefabricated abutments were used:

- Groups C1–C3: SICvantage Bonding Base CAD/CAM red, straight, CEREC for the internal conical connection, height: 4.65 mm (SIC Invent AG) (Figure 2A).
- Group H: SIC Bonding Base CAD/CAM, straight, CEREC for the internal hex connection group, height: 4.65 mm (SIC Invent AG) (Figure 2B).

**Figure 3:**
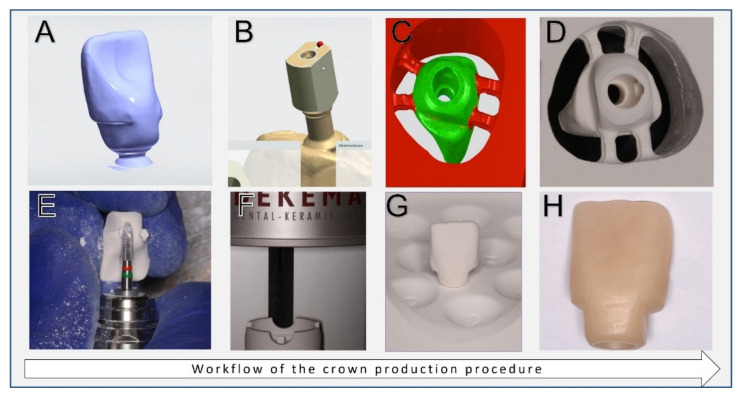
CAD/CAM crown design and production. (**A**) The scanned prototype. (**B**) The scanned scan body. (**C**,**D**) Access canal for the retention screw. (**E**) Crown finishing. (**F**,**G**) Sintering the crowns. (**H**) The final crown.

Thirty-two anatomical monolithic zirconia crowns mimicking the first upper right incisor with a length of 12 mm and a width of 8 mm were customized from 3 mol% yttria-stabilized tetragonal zirconia polycrystal discs (Dental Direkt GmbH, Spenge, Germany). In addition, two nooks were designed in the cervical (mesial and distal) area for a precise grip of the pull-off instrument. The predesigned crown prototype was sent to a milling center (Cadspeed, Nienhagen, Germany), where it was scanned (D1000, 3Shape, Copenhagen, Denmark) and optimized in a design program (Trios Design Studio, 3Shape, Copenhagen, Denmark) (Figure 3A). For this purpose, a scan body was connected to the respective implant so that the crown and the implant could then be matched (Figure 3B). The crowns were milled using a 5-axis dental milling machine (DGSHAPE DWX-52DCI, Roland DG Corporation, Shizuoka-ken, Japan). The crowns were designed and fabricated for screw retention on the implants (Figure 3C,D). After milling, a specific finishing bur was used to separate the crowns from the blank (Figure 3E). After that, the crowns were inserted in a high-temperature sintering furnace (Dekema, Dekema Dental GmbH, Freilassing, Germany) (Figure 3F,G). All sintered crowns were glazed to attain a smooth surface following the manufacturer instructions (Figure 3H).

The bonding surface of the crowns and the CAD/CAM abutments was air-abraded by applying 50 µm Al_2_O_3_ particles with 0.5 bar pressure. To cement the crowns onto the titanium abutments, a Multilink^®^ Hybrid Abutment composite cement (Multilink Hybrid Abutment, Ivoclar Vivadent, Schaan, Liechtenstein) was employed.

**Figure 4:**
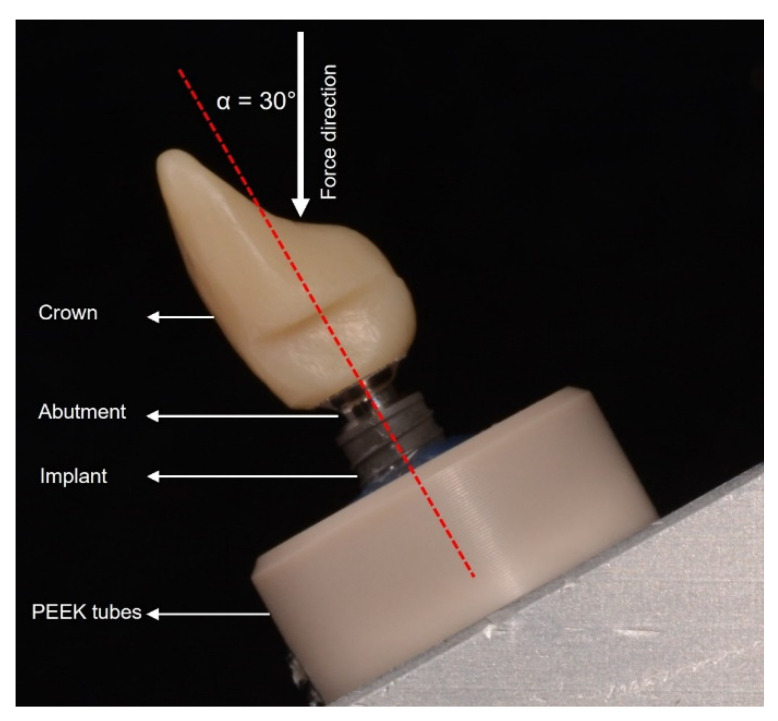
Standardized photograph showing one sample before chewing simulation. The red dotted line represents the long axis of the implant and the crown.

All implants were hosted in polyether ether ketone (PEEK) tubes using a dual polymerizing acrylic resin (LuxaCore^®^ Dual, DMG, Hamburg, Germany). To ensure the ISO 14,801 requirements, a distance of 3 mm of the upper part of the implant was exposed to mimic marginal bone loss. All implants were mounted perpendicularly into the PEEK tubes using a special securing device. The hosting resin had a modulus of elasticity between 8.7 and 10.3 GPa [27]. For the dynamic loading test, all specimens were positioned at an angle of 30 degrees from the vertical axis (Figure 4). Unified images were used to determine the embedding angle and lever arm for each of the 32 samples (Affinity Designer 1.10.4, Serif (Europe) Ltd., Nottinghamshire, UK) (Figure 4).

The crown–abutment assemblies were connected to the implants in four different manners:

Group C1: The abutments were screwed onto the respective implants with a torque value of 20 Ncm, as suggested by the manufacture. The screws were kept until the end of the dynamic loading and were removed before the pull-off test. The screws (SIC Standard, SIC Invent AG) were made of titanium grade 5 with a length of 9.5 mm and a diameter of 1.9 mm (head) and 1.6 mm (shank and thread).

**Figure 5:**
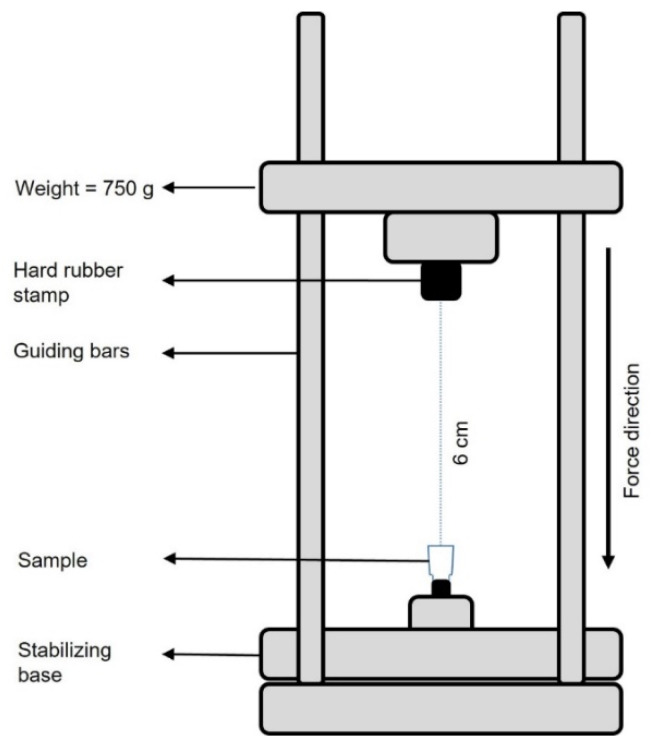
Schematic diagram of the tapping device.

Group C2: Screwless connection: To ensure a standard fixation method of the crown–abutment assemblies to the implants, a special loading force device was used to tap/knock in the samples of this group in a standardized way (Figure 5). This device was carrying a weight of 750 g, which was released onto the samples from a vertical direction and a distance of 6 cm. This procedure was carried out three times for each sample [28].

Group C3: All specimens of this group were assembled with a screw using the preloading torque recommended by the manufacturer (20 Ncm) to ensure the maximal anchorage of the abutments into the implants. Then the retention screws were removed before the chewing simulation.

Group H: A screw-retained control group with an internal hexagonal implant–abutment connection was used. The screws were tightened (20 Ncm) according to the manufacturer’s recommendation.

**Figure 6:**
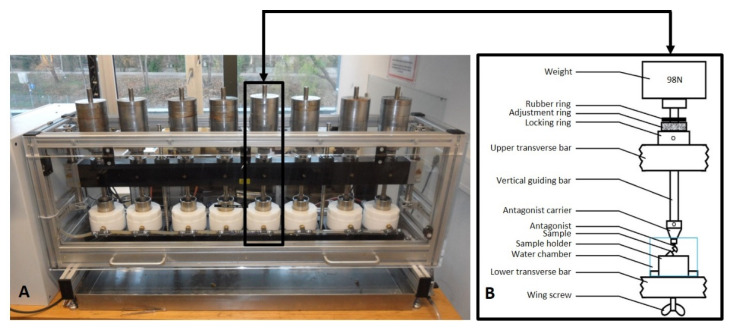
(**A**) The assemblies mounted in the artificial mastication machine CS-4.8 (SD Mechatronik, Feldkirchen-Westerham, Germany). (**B**) Schematic drawing of the test chamber.

All samples (*n* = 32) were subjected cyclic mechanical loading in a mastication simulator (Type CS-4.8, SD Mechatronik, Feldkirchen-Westerham, Germany), including water thermocycling (5 to 55 °C) [29,30]. A load of 98 N was applied onto the assemblies in both vertical (1.5 mm) and horizontal (0.5 mm) motions to emulate the physiological masticatory cycle (Figure 6A,B). During dynamic test, specimens were checked by one of the authors (VW) two times a day to discover any complication. Permanent deformation or fracture of any of the pieces was defined as failure in this study. The assemblies were aged for 10 million loading cycles (simulation of approximately 40 years of clinical service). The settings of the artificial mastication machine applied in this study are summarized in Table 1.

**Figure 7:**
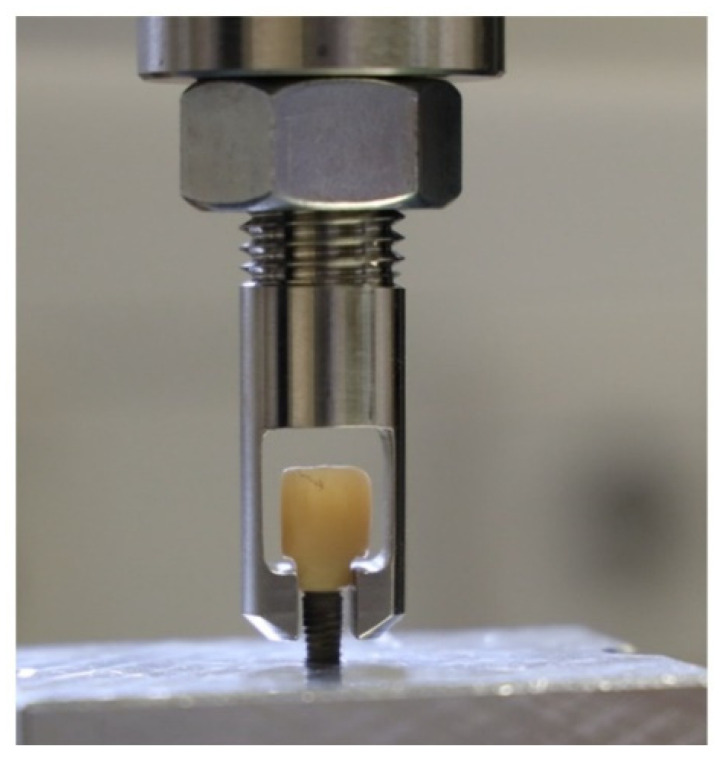
Mounting of the samples in the universal testing machine and with the pull-off force clamp in situ.

After the dynamic loading, all samples were visually assessed using a light microscope (LM) (Olympus Stereo Microscope SZH10, Olympus, Tokyo, Japan) at 7-fold magnification. To grant observing the samples for any surface deformations therearound, equidistantly at the area of the implant–abutment connection under the light microscope, a special apparatus was equipped according to a previously described procedure [16]. As shown in Figure 7, all samples surviving the dynamic loading testing after the microscopic investigations were firmly attached into a universal testing machine (Zwick Z010/TN2S, ZwickRoell GmbH, Ulm, Germany) for monotonic failure/pull-off force testing. To ensure that the pull-off force was applied perfectly axial to the long axis of the implant, each implant was screwed in and firmly held in a customized metal base. All specimens and the bases were attached to the universal testing machine, and a vertical pull-off force was applied by gripping the samples at the cervical nooks (Figure 7). The test machine speed was set to 10 mm/min for all samples, and the pull-off force was applied until the abutments were detached from the implants or the crowns were decemented from the abutments. A graphical illustration was obtained after each trial using the Zwick testXpert^®^ V 7.1 (Zwick) software (Zwick testXpert, ZwickRoell GmbH, Ulm, Germany) to record the pull-off force of each assembly (measured in N).

### 2.2. Statistical Analysis

For a descriptive analysis of the monotonic failure strength, the mean, median, and standard deviations of the acquired data were computed and statistically analyzed at the Institute of Medical Biometry and Medical Statistics of the Albert Ludwig University, Freiburg, Germany (KV). Boxplots were used for a graphical explanation of the data. A one-way analysis of variance was used to check for differences among the groups for monotonic failure pull-off force results. The *p*-values for subsequently pairwise comparisons were corrected using the Students–Newman–Keuls method in order to correct for multiple testing. Statistical significance was set at *p* < 0.05. All obtained data were statistically analyzed using the software STATA (STATA version 17.0; StataCorp LLC, College Station, TX, USA).

## 3. Results

### 3.1. Results from the Dynamic Loading (Chewing Simulation)

All specimens survived the chewing simulation without any evidence of complications. This yielded an overall implant and superstructure survival rate (screwed and screwless groups together) of 100%.

Hence, no differences in survival between the different retention method groups (screwed and screwless) were observed.

According to the light microscope assessment, all specimens exhibited no postloading defects at the implant or abutment level. However, all specimens (100%) of group C1 (screw-retained conical connection) showed screw loosening. The group H (screw-retained hexagonal connection) did not show screw loosening after the dynamic loading. This was rated by controlling the screw retention force using a torque gauge before the pull-off test. All the specimens of group H did not show any loss of the preloading torque (20 Ncm). Decementation of zirconia crowns was detected during the dynamic loading in 4 of the 32 specimens (12.5%) (2 specimens in C2 at 9.9 million cycles and 1 specimen each in group C1 at 10 million cycles and C3 at 8.5 million cycles). The incidence rates of complications for the different groups are summarized in Table 2.

### 3.2. Quasi-Static Pull-Off Retention Stability Test Results

After the specimens were submitted to pull-off tests in the universal testing machine, they were examined to characterize the failure mode.

**Figure 8:**
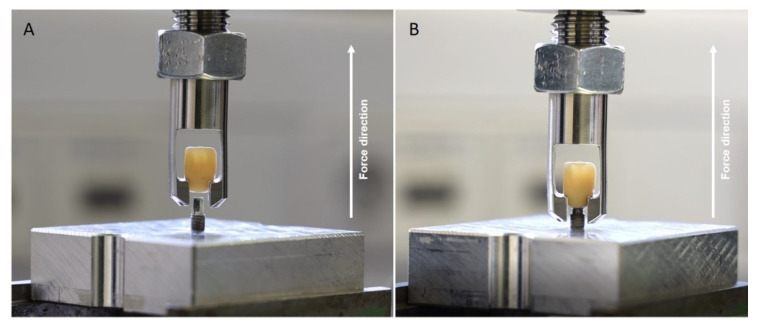
Detachment of the IAC assemblies during the pull-of leading to failure: (**A**) Sample from group H showed decementation; (**B**) sample from group C3 showed pull-off of the abutment.

After quasi-static pull-off, all specimens exhibited either detachment of the abutment from the implant or retention loss (decementation) of the crown from the abutment (=failure; Figure 8). No catastrophic failure (=abutment or implant fracture) was observed. The means and standard deviations of the pull-off forces leading to failure for the various groups are listed in Table 3. One specimen from group C2 was lost and had to be excluded from the statistical analysis.

**Figure 9:**
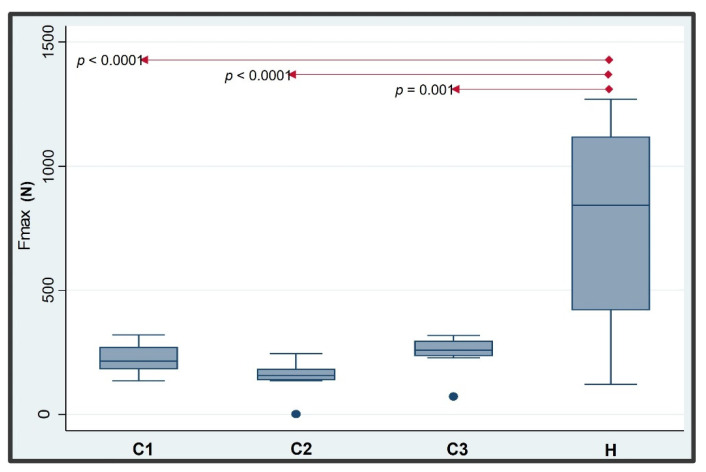
The boxplot diagram demonstrated the pull-off failure loads of the groups (*n* = 31). Horizontal red lines represented statistically significant differences between the four groups.

One sample of group C1 showed a crown decementation at a force of 166 N. This sample was recemented and subjected to a pull-off test again. The subsequent pull-off experiment led to a disassembling of the abutment from the implant at 9 N. Therefore, we considered the value of 166 N as the more correct value for analysis. A sample of group C3 similarly showed a crown decementation at a force of 78 N. The second pull-off test after recementation led to a disassembling of the abutment from the implant at 3 N. We considered also here the higher value (78 N) for the statistical analysis. The mean monotonic pull-off failure forces (±SD) ranged from 171.6 N (±37.7 N) for group C2 to 769.9 N (±413.5 N) for group H. The differences were statistically significant between the four groups (*p* < 0.0001; Figure 9). Significantly higher pull-off forces were found for the butt–joint group as compared with the conical connection groups. Pairwise comparisons demonstrated a statistically significant difference between group H and all groups C. No statistically significant differences were found between the screwed and screwless conical IAC groups (Figure 9).

## 4. Discussion

This in vitro study evaluated the (retention) stability of two implant–abutment connection (screwed vs. screwless) designs in an in vitro environment using artificial mastication, followed by quasi-static pull-off test. The current study found that the presence or absence of the retention screw during chewing simulation has no influence on the stability of the Morse taper conical implant–abutment complex while being artificially loaded. Moreover, internal hexagonal or internal conical connected implant–abutment geometries restored with monolithic zirconia crowns exhibited an equivalent performance during chewing simulation (i.e., no failure). In the present investigation, no implant fracture in both, the hexagonal and/or the conical connection, groups was reported during the chewing simulation. Although a systematic review on the clinical application of different implant–abutment connections did not find significant differences in failure rates between different connections [8], oral implants with conical connections seem to be more stable during artificial loading [15,16].

In the control group, all cemented crowns came loose from the corresponding abutments during the pull-off test at a mean pull-off value of 770 N. In a recent study [31], the authors investigated different crown–abutment cements and observed a mean pull-off force of 820 N for the group cemented with a permanent composite cement (SpeedCEM^®^ Plus). In that investigation, the abutment height was 5 mm, whereas it was 4.65 mm in our investigation. The results of both studies, therefore, are very comparable. In no instance did an abutment–implant dislocation occur in our investigation.

However, only two samples of the C groups showed decementation of the crown from the abutment before the abutment was detached from the implant. For these two specimens, the friction between the abutment and the implant was higher than the pull-off force required to decement the crown. Possibly, there was an inaccuracy during the cementation process.

On the implant level, our results contrasted an in vitro study by Ugurel et al. (2015), which reported that the mechanical strength of the screwless Morse taper implant–abutment connection is lower than that of the screw-retained ones [32]. The authors of that study reported that all samples of the screwless Morse taper (3°) implants failed to survive the planned dynamic loading (1.2 × 106 loading cycles). All implants fractured at less than 100,000 loading cycles when a load of 120 N was applied at 30° to the long axis of the implants. In our in vitro study, all screwless Morse taper implants survived the 10 million cycles with a load of 100 N. This variance between studies is astonishing, since both studies applied comparable loading conditions and both implants had almost identical implant diameter and cone angles. However, the implants in the investigation of Ugurel et al. (2015) obviously had a smaller thickness of the implant head wall, which was in contact with the abutment, which was not as resistant as the implant heads of the present implant system. The screw-retained internal hexagonal groups in the study of Ugurel et al. (2015) exhibited early abutment and/or screw fractures. Those results were also different compared with the screw-retained implant group in our study during the chewing simulation.

The most common technical complications of implant-supported prosthetic reconstructions are screw loosening or screw fractures [8,33,34,35,36]. Pjetursson and his team (2018) reported an overall screw loosening event of 6% after an observation period of 5 years. The external connections exhibited a higher prevalence of screw loosening than internal connections [8]. Another review showed that screw loosening or screw fractures varied between 2% and 45% of the implant restorations, with a high incidence in single implant-supported restorations [37]. Screw loosening is affected by many factors, including screw design, screw material, screw diameter, retention torque, and implant–abutment connection design [38]. In the present study, implants with an internal hexagonal connection did not show screw loosening after 10 million chewing cycles, whereas implants with a screw-retained conical abutment connection showed 100% of screw loosening. A torque loss of the screw-retained conical implant–abutment connections ranged between 27.6% and 40.9% in previous studies [39,40,41]. Screw loosening of the conical connection group during the chewing simulation might be the result of the abutment “sinking” into the implant because of the chewing load. Studies have reported a vertical displacement of the abutment in relation to the implant [39,42,43]. This vertical displacement might cause slippage between the threads of the screw and the threads of the implant, resulting in the loss of the preload and screw loosening.

On the other hand, the aforementioned vertical displacement seems to have a positive effect on the pull-off force required to detach the abutment from the implant in the conical connection groups. Our results revealed a mean pull-off force ranging from 172 to 246 N for the screwless conical implant–abutment connections without statistically significant differences between the three groups C1 to C3. Pull-off forces were previously reported with a mean value of 120 N [44], 77.6 N [39], and 51 N [41] for the tested (conical) groups when Zimmer Biomet, Ankylos C/X, and Blackfix implant systems were used, respectively. The difference in the pull-off forces between our results and the results of the former three studies might be associated with the number of loading cycles and loading weights applied. Kofron et al. (2019) loaded the samples in 1000 cycles by applying a load of 200 N, Hsu et al. (2018) loaded the samples for 1 million cycles with a cyclic load of 18 to 180 N, and Pintinha et al. (2013) loaded the samples for 500,000 cycles with a load of 100 N. Their results, together with our results, indicated an increase in the pull-off force possibly due to the increased friction (cold welding) between the abutment and the implant.

Another influencing factor besides the loading protocol might be the implant–abutment connection geometry. The implants used in the first study [44] were characterized by a cone angle of 1° (Tapered Screw-Vent, Zimmer Biomet, Warsaw, IN, USA). The implants evaluated in the second study [39] had a cone angle of 5.7° (Ankylos C/X, Dentsply-Friadent, DENTSPLY-Friadent GmbH, Mannheim, Germany), whereas implants with a cone angle of 11° (Blackfix, TitaniumFix, São José dos Campos, Brazil) were tested in the third study [41]. In the current investigation, the implants had an internal cone angle of 2.8°. According to Hsu and his team (2018), implant–abutment connections with smaller cone angles might result in increased vertical displacement and, subsequently, in an increase in the friction and, finally, in the pull-off force. Moreover, the tap-/plug-in methods (applying a screw torque or tapping the superstructure into the implant) did not reveal a significant difference of the pull-off forces and of the mechanical stability of the conical implant–abutment connections. In this regard, it is noteworthy to mention that the pull-off force required disassembling the abutment, and the implant in case of the plug-in method (applying a screw torque) might be increased if the screws were repeatedly activated as reported by a recent in vitro study [45]. However, the results of the utilized implant system in the current study point to the possible elimination of the retention screw for the attachment of the prosthetic restoration. This result allows the expansion of options for clinicians to overcome improper implant positions and/or angulations in order to avoid the screw access opening in the anterior zone to be visible. A crown could be cemented extraorally to the respective abutment, and this assembly inserted via tapping into the implant.

A standardized test protocol, as utilized in the present investigation, seems to be helpful for comparing different implant materials and implant designs [46]. In the present study, the implant-related features were kept invariable for the test groups, while the suprastructure retention method (screwed attachment, tapped-/knock-in attachment) was the variable for the experiment. A standardized loading situation was applied to all test specimens, which produced commensurate results. However, the significance of the current study may be limited due to a low sample size of only eight specimens per group.

In summary, all but one implant and restoration survived the long-term artificial loading, irrespective of the abutment retention method or implant–abutment connection design. This indicates that the screwless implant–abutment connection, irrespective of the assembling method, is a reliable option for the presented implant system. However, this should be clinically approved. Moreover, despite the lower pull-off force required to disassemble the abutment from the implant in case of the Morse taper screwless implant abutment connection, this force remains in a clinically acceptable range.

## 5. Conclusions

The survival rates of screw-retained and screwless abutments used in the current in vitro study are similar. The use of a screwless Morse taper implant–abutment connection represents a valid form of treatment for single-tooth replacement. Screw loosening in a Morse taper implant–abutment connection was frequent but had no effect on the (retention) stability of the connection. Nevertheless, the screwless implant–abutment connections used in our study seem to withstand average occlusal forces even after an extended interval of artificial loading.

## Figures and Tables

**Table 1:** Settings of the chewing simulator.

Specifications of the Chewing Simulator	
Total Number of cycles	10 Million
Movement	Vertical: 1.5 mm; horizontal: 0.5 mm
Speed and frequency	Descending: 60 mm/s; rising: 60 mm/s; forward: 55 mm/s; backward: 55 mm/s; frequency: 1.9 Hz
Weight	10 kg (98 N)
Thermocycling	Cold: 5 °C; hot: 55 °C

**Table 2:** The incidence rates of each complication in the different groups (IAC = implant–abutment connection).

IAC Design	Hexagonal IAC	Conical IAC		
Group	H	C1	C2	C3
Prevalence of implant fractures	0	0	0	0
Prevalence of abutment fractures	0	0	0	0
Prevalence of crack/deformation events	0	0	0	0
Prevalence of screw loosening events	0	8	0	0
Prevalence of crown–abutment decementations	0	1	2	1
Implant survival rates	100%	100%		
Abutment survival rates	100%	100%		
Overall survival rate	100%			

H = implants with internal hexagonal connection; C1, C2, C3 = implants with internal conical connection.

**Table 3:** Mean values for failure loads (pull-off forces) of the test groups (N = 31).

Group	No.	Max (N)	Min (N)	Mean (N)	SD (N)	No. of Crown–Abutment Decementations	No. of Abutment–Implant Detachments
C1	8	321	136	224.4	61.9	1	7
C2	7	245	136	171.6	37.7	0	7
C3	8	318	72.7	247.0	77.4	1	7
H	8	1270	121	769.6	413.5	8	0
